# Plasma and serum BDNF differentially relate to fNIRS prefrontal cortex activity during executive function and memory tasks

**DOI:** 10.1016/j.brainres.2025.149827

**Published:** 2025-07-08

**Authors:** Flaminia Ronca, Cian Xu, Tom Gurney, Antonia Hamilton, Giampietro Schiavo, Dennis Chan, Ilias Tachtsidis, Paola Pinti, Paul W. Burgess

**Affiliations:** a Institute of Sport, Exercise and Health, Division of Surgery and Interventional Sciences, Faculty of Medical Sciences, University College London, London, UK; b Institute of Cognitive Neuroscience, Psychology and Language Sciences, Faculty of Brain Sciences, University College London, London, UK; c Department of Neuromuscular Diseases and UCL Queen Square Motor Neuron Disease Centre, UCL Queen Square Institute of Neurology, University College London, London, UK; d UK Dementia Research Institute, University College London, London, UK; e Department of Medical Physics and Biomedical Engineering, University College London, London, UK; f Department of Psychological Sciences, Birkbeck, University of London, London, UK

**Keywords:** Hemodynamic, Neurotrophic, Cognition, Reaction time, Inhibition, Attention

## Abstract

**Introduction:**

Brain-Derived Neurotrophic Factor (BDNF) is known to play significant roles in memory through synaptic plasticity and hippocampal volume. However, its function in the prefrontal cortex remains less explored, and differences in the associations of plasma BDNF (pBDNF) and serum BDNF (sBDNF) with cortical function are at present poorly understood. Therefore, this study aimed to investigate whether there are differential relationships between pBDNF and sBDNF with prefrontal cortex activity during executive function and episodic memory tasks.

**Methods:**

Twenty-three participants (7 females, age 32 ± 15 years) provided venous blood samples and completed executive function and episodic memory tasks at three time points (weeks 0, 6, and 12). Prefrontal cortex function was additionally measured using functional near-infrared spectroscopy (fNIRS) in week 6.

**Results:**

There was a significant positive association between pBDNF (but not sBDNF) and episodic memory performance (p < 0.05), and with higher CBSI-beta in the left orbitofrontal and frontopolar regions (R range = 0.41 to 0.58; p range = 0.004 to 0.049) during both encoding and retrieval blocks. During inhibition and attention tasks, pBDNF correlated with lower CBSI-beta in the medial frontopolar and orbitofrontal regions (R range = -0.41 to −0.55; p range = 0.007 to 0.05) but did not predict cognitive performance. In contrast, sBDNF was associated with higher CBSI-beta across the prefrontal cortex during endogenous attending only, but was not associated with performance on any task.

**Discussion:**

Better cognitive performance and higher neural activity during episodic memory suggest that pBDNF may be involved in supporting prefrontal cortex functions for episodic memory. Different associations between pBDNF and sBDNF and both cognitive and imaging outcomes may reflect the differing bioavailability of these two BDNF pools, with implications for future mechanistic studies.

## 1 Introduction

Brain-Derived Neurotrophic Factor (BDNF) is a neurotrophin involved in the growth, maintenance and survival of neurons. It is well established for its ability in supporting memory functions, particularly through its influence on synaptic plasticity, and hippocampal volume and function (Erickson et al., 2010; Van Praag, 2009). However, its role in executive functioning remains less explored, and the existing literature currently presents contradicting results. Understanding whether a relationship with executive functioning exists could provide insights into how BDNF supports complex higher-order cognitive processes beyond memory, such as planning, decision-making, and inhibitory control.

Circulating BDNF levels can be derived from plasma or serum, and these are sometimes used interchangeably in the literature, even though they are reflective of distinct pools of BDNF with different bioavailability. Plasma BDNF (pBDNF) represents small concentrations of freely circulating levels of this neurotrophin, which are immediately bioavailable and able to cross the blood–brain-barrier. Plasma, but not serum, BDNF has been shown to correlate with hippocampal and prefrontal cortex levels of BDNF in several animal models (Klein et al., 2011) and with cerebrospinal fluid concentrations of BDNF in humans (Pillai et al., 2010). In contrast, serum BDNF (sBDNF) reflects the majority of the neurotrophin’s concentration synthesised by the body, which is stored in platelets and released upon sample clotting. This makes sBDNF more likely to reflect BDNF transcription across the body, but it also makes it susceptible to interactions with inflammatory status which impact platelet activity (Gejl et al., 2019; Pedersen et al., 2017).

Despite such mechanistic differences, at present there is a dearth of research exploring how these two pools of BDNF might differentially relate to cortical brain function, particularly during executive function tasks. There is already a wealth of literature that demonstrates a relationship between BDNF and various aspects of memory (Shimada et al., 2014; Erickson et al., 2010; Komulainen et al., 2008), which is theoretically underpinned by BDNF’s role in synaptic plasticity, synaptogenesis and neurogenesis (Choi et al., 2018; van Praag et al., 2000). However, its potential role in higher order processes that are crucial to managing and regulating behaviour, such as executive function, remains less clear, as the literature presents conflicting results. For example, cross-sectional studies have reported no correlation between executive function performance and pBDNF (Komulainen et al., 2008) or near-significant trends with sBDNF in the elderly (Shimada et al., 2014). Others report a mediating effect of sBDNF on executive function in obesity (Si et al., 2021; Kaur et al., 2016), and some intervention studies report an effect of increasing s/pBDNF on improving executive function (Leckie et al., 2014; Wagner et al., 2019). However, this literature has so far focused on behavioural data in the elderly or in clinical populations, it has evaluated different subdomains of executive function, and has measured either sBDNF or pBDNF, complicating comparison across the various reports. Further to this, while most imaging studies have focused on deep brain structures such as the hippocampus (Erickson et al., 2010), there is still a lack of knowledge on the specific effects of BDNF on the prefrontal cortex, particularly in heathy individuals. Due to the dysregulation of BDNF in clinical populations, and to the crucial role of the prefrontal cortex in regulating behaviour in specific circumstances, such as depression or dementia, the interpretation of its mechanistic effects is not always translatable between studies in healthy and clinical groups (Angoa-Perez et al., 2017; Miranda et al., 2019). Therefore, it is important to first explore these effects in healthy adults to understand how s/pBDNF might relate to different subdomains of executive function and region-specific cortical hemodynamics in the frontal lobe.

Therefore, the aim of this study was to explore whether peripheral levels of plasma and serum BDNF related to different subdomains of executive function and episodic memory, and to associated prefrontal cortex hemodynamics measured through functional Near Infrared Spectroscopy (fNIRS). To better understand BDNF’s role in prefrontal cortex hemodynamics, executive function tasks were included due to the prefrontal cortex’s direct involvement in attention and inhibition; while short-term episodic memory was included because of its supporting role during encoding and retrieval processes, and due to the established relationship between memory and BDNF previously observed through deeper brain structures. We hypothesised that pBDNF would be more likely to show a relationship with prefrontal cortex activity than sBDNF given its greater bioavailability and ability to cross the blood–brain-barrier. In the exploration of different tasks, we hypothesised that episodic memory and executive function tasks would exhibit regional differentiations of brain hemodynamics in relation to BDNF.

## 2 Methods

### 2.1 Participants

Participants were included in the study if they were 18–60 years old, classified as sedentary (< 30 min of moderate-intensity exercise for <3 days/week for the past three months), but otherwise healthy and not presenting with any cardiovascular or neurological illnesses. Participants provided written informed consent prior to taking part in the study. Ethical approval was obtained in line with the Declaration of Helsinki from the UCL Research Ethics Committee (ID: 21745/002).

### 2.2 Study design

Participants attended the laboratory on three visits at week 0, 6, 12, always between 8am at 10am and in a fasted state. At each laboratory visit, participants provided a venous blood sample and completed a battery of cognitive tasks. In week 6, after participants had already been exposed to the cognitive battery once (in week 0) and were therefore not naïve to the tasks, brain function was also measured via functional near-infrared spectroscopy (fNIRS).

### 2.3 Blood sampling

At each visit, participants provided venous samples from the cephalic vein (8 ml), of which 4 ml were collected into an ethyl-enediaminetetraacetic acid (EDTA) tube and 4 ml into a serum tube. The EDTA samples were immediately centrifuged at 4000 rpm for 10 min at 4 °C. The serum samples were left at room temperature for 30 min before centrifugation at 3500 rpm for 10 min in line with the protocol described by Gejl et al. (2019). After centrifugation, the plasma and serum were separated into two cryovials and immediately frozen at −80 °C for future analysis. All samples were thawed and analysed together at the same time, 3 months after study completion. Plasma and serum BDNF were measured by single molecule array (SIMOA) on an HD-x analyser (Quanterix), according to manufacturer’s instructions.

Plasma and serum BDNF were measured by single molecule array (SIMOA) on an HD-x analyser (Quanterix), according to manufacturer’s instructions. Briefly, samples were thawed at 21 °C, and centrifuged at 10,000 RCF for five minutes at 21 °C. On-board the instrument, samples were diluted 1:500 with sample diluent and bound to paramagnetic beads coated with a capture antibody specific for human BDNF. BDNF bound beads were then incubated with a biotinylated BDNF detection antibody in turn conjugated to streptavidin-β-galactosidase complex that acts as a fluorescent tag. Subsequent hydrolysis reaction with a resorufin β-D-galactopyranoside substrate produces a fluorescent signal proportional to the concentration of BDNF present. Singlicate measurements were taken of each sample. Sample concentrations were extrapolated from a standard curve, fitted using a 4-parameter logistic algorithm. Intra-assay and inter-assay CVs were less than 10 % and 15 % respectively, as determined by 16 quality controls.

### 2.4 Cognitive battery

**Fig. 1:**
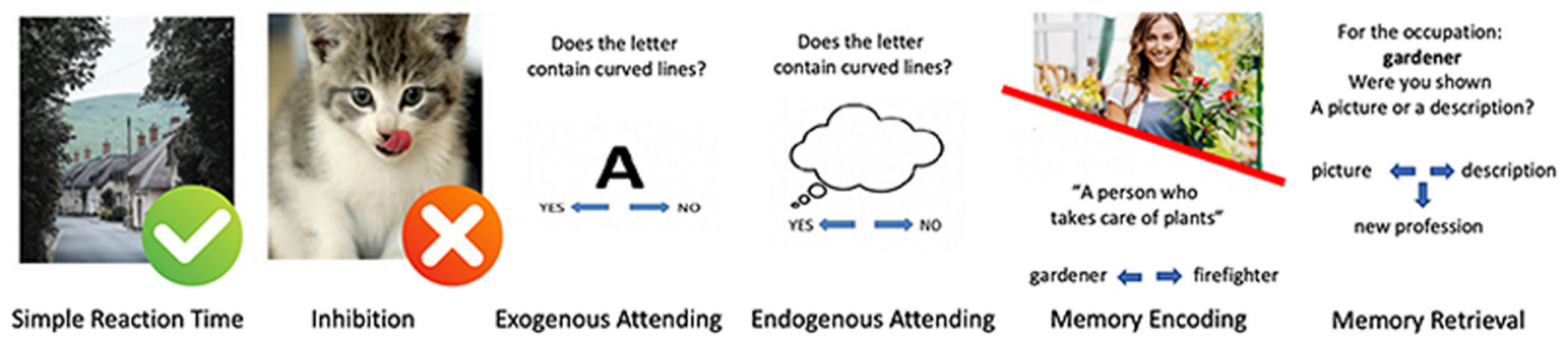
Battery of cognitive tests in the sequence it was administered (Crum et al., 2023; 2024). The simple reaction time task required pressing the spacebar on a desktop keyboard every time an image of a landscape or animal appeared. The inhibition task also presented images of landscapes and animals, but required withholding a press of the spacebar if an image of a kitten appeared. The exogenous attending task presented letters of the English alphabet in sequence, participants had to indicate if the letter shown contained curved lines or not. The endogenous attending task required the same responses, but letters of the alphabet were not shown on the screen, the participant was asked to imagine the letters in the correct sequence. The memory encoding task presented either a picture or a description of a profession. After all encoding stimuli were shown, the source memory retrieval task then asked participants if they were shown a picture or description of each profession, or neither. The full battery took less than 15 min to complete.

The executive function battery was conducted as described in Crum et al. (2022) in a single block design, with average block duration of 30–40 s. Briefly, the battery included six blocks, including one task each (Fig. 1). The first two blocks consisted of simple reaction time and inhibition tasks using photographs as stimuli, widely known and implemented as Go/NoGo tasks (Eigsti et al., 2006; Zhang et al., 2017). The next two blocks consisted of exogenous and endogenous attending tasks which implemented the alphabet as stimuli, also known as the Alphabet Task, designed to measure one’s ability to maintain stimulus-oriented vs stimulus-independent modes of attending (Gilbert et al., 2005; Burgess et al., 2007; Burgess and Wu, 2013). The two final blocks measured episodic memory and consisted of source memory encoding and retrieval (Turner et al., 2008), these implemented photographs and written descriptions of people and their professions as stimuli. Mean reaction times for all tasks were calculated using only correct responses, while errors were calculated as total number of errors. The full battery, its development, processing and stimuli are described in detail Crum et al. (2022, 2024).

### 2.5 fNIRS brain imaging

**Fig. 2:**
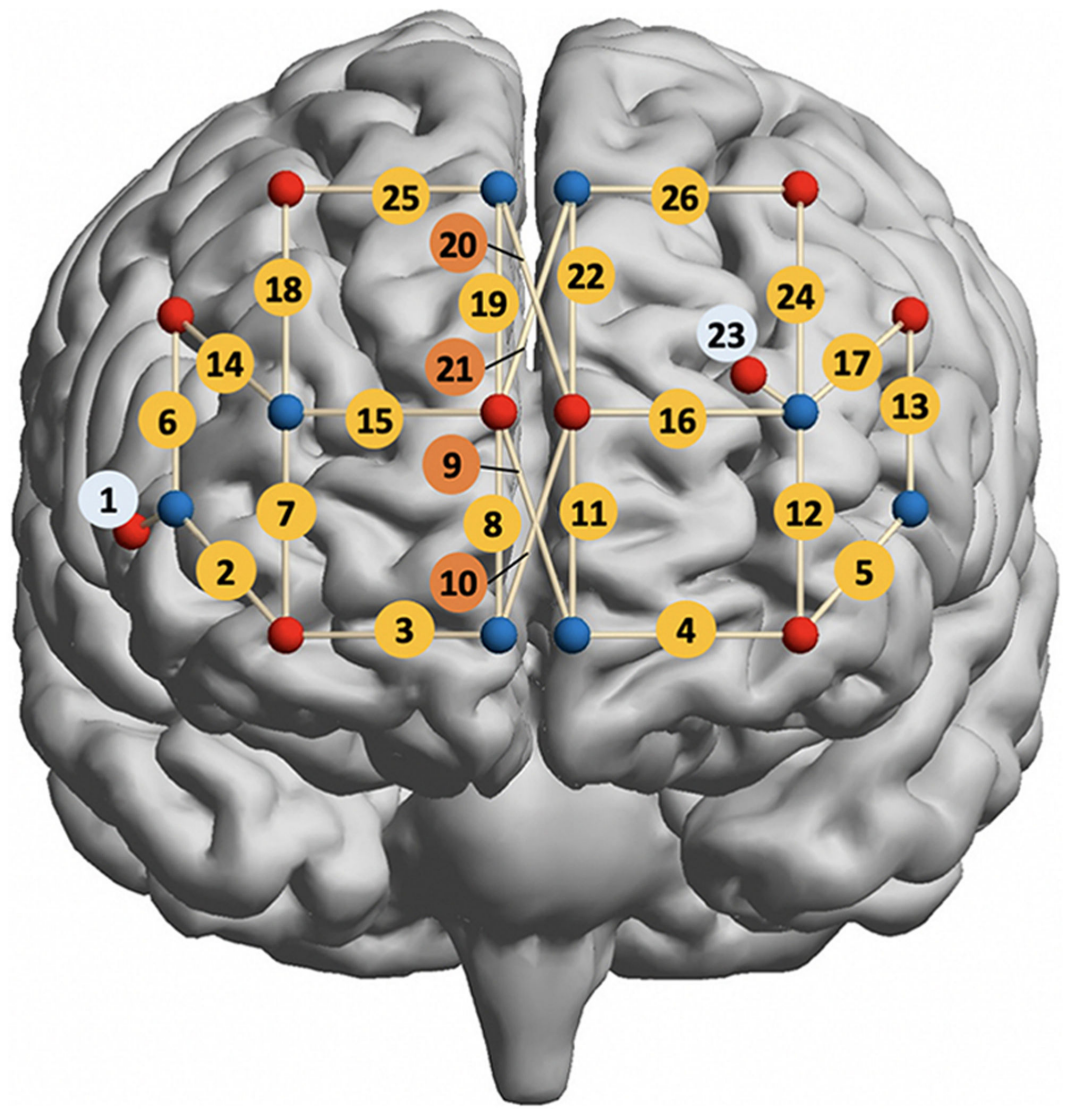
Channel location for the fNIRS Artinis system. Yellow circles indicate channel numbers for the 30 mm channels and orange circles indicate 4 longer channels (32 mm distance) positioned to cover medical prefrontal cortex. Pale blue circles indicate the two short channels 01 and 23 (10 mm). Frontopolar optodes were positioned in such a way that four channels could be derived by four optodes through both diagonal and vertical cross-over (channels 08 – 11 and 19 – 22). See Supplement 1 for full channel coordinated and locations.

Brain activity in the prefrontal cortex was measured during cognitive testing at 25 Hz using a mobile fNIRS device (Brite MKII, Artinis Medical Systems BV, Netherlands) equipped with 10 light sources and 8 detectors, yielding 24 long channels (30–32 mm inter-optode distance) and 2 short channels (10 mm) (Fig. 2). The two short channels were located bilaterally in the prefrontal area in accordance with suggested placements (Sato et al., 2016; Wyser et al. 2020) in order to eliminate interferences caused by haemodynamic variations observed in the superficial tissue layers and to improve the selectivity to the brain. The four longer channels, located on the frontal pole, were generated by two sets of sources and one set of detectors, symmetrically distributed vertically on either side of the frontal pole, with a minimum inter-optode distance of 10 mm. This arrangement is dictated by the existence of the central longitudinal fissure, and was chosen to monitor brain hemodynamics from either side of the frontal pole (BA10), allowing to investigate any lateralisation within medial prefrontal cortex. Data quality checks were carried out using QT_NIRS (htps://github.com/lpollonini/qt-nirs).

A 3D magnetic digitiser (Patriot, Polhemus, Vermont, USA) was used to record optodes’ coordinates for each participant before cognitive testing. Implementation protocols were based on 10–20 EEG system and 5 selected anatomical landmarks (Central zero, Nasion, Inion, right- and left-auricular points,) for orientation. The coordinates were registered onto the standard brain template (MNI152) using the NIRS_SPM package (Ye et al., 2009) and spm8 (Penny et al., 2011). The estimated channel MNI coordinates, corresponding anatomical labels and Brodmann area (BA) can be found in Supplement 1.

The fNIRS data pre-processing was conducted in accordance with the standardised pipeline illustrated by Pinti et al. (2019), explained in detail in Supplement 2. The modified Beer-Lambert Law was employed to calculate the relative changes in oxyhaemoglobin (HbO_2_) and deoxyhaemoglobin (HbR), which were then combined using the correlation-based signal improvement (CBSI) method (Cui et al., 2010; Tachtsidis & Scholkmann, 2016). Channel-wise general linear models (Friston et al., 1994) were fit to the pre-processed signals. The design matrix included task regressors computed through the convolution of the task blocks of each condition (simple reaction time, inhibition, endogenous attending, exogenous attention, memory encoding, memory retrieval) with the canonical hemodyamic response function (HRF) as well as the heart rate, respiration rate (Biopac MP160 station and RSPEC-R modules with AcqKnowledge 5.0, at 2000 Hz), head accelerometery (built into the Brite MKII unit), and the nearest short channel as additional regressors. Single-subject CBSI beta values were obtained through the SPM-fNIRS toolbox for the following contrasts of interest: Simple reaction time > 0, Inhibition > 0, Endogenous attending > 0, Exogenous attending > 0, Memory encoding > 0, Memory retrieval > 0. These are an indication of the presence of brain activity in response to the task conditions and were used in all further correlation analyses.

### 2.6 Data analysis

Data analysis of fNIRS beta values and of the behavioural data was conducted using RStudio (R Core Team, 2022). All data was checked for normality through visual inspection and Shapiro-Wilk tests, and BoxCox transformed where appropriate. Alpha levels were set to 0.05 for all analyses. To understand whether s/pBDNF were associated with cognitive performance, linear mixed models with repeated measures were conducted to predict performance on each cognitive task (reaction times and errors), where sBDNF or pBDNF were inputted as predictors in separate models. All models controlled for age and sex, with week*BDNF interactions (to account for possible changes in BDNF levels by visit), and random effects per participant. To understand the relationship between BDNF and brain activity, Spearman and Pearson correlations were conducted between s/pBDNF values and CBSI beta values for each channel from the fNIRS imaging data. P values for these correlations are presented without correction for multiple comparisons. For completeness, paired Wilcoxon tests were also conducted within pairs of cognitive task contrasts for this purpose (simple reaction time – inhibition, exogenous – endogenous attending, encoding – retrieval).

## 3 Results

### 3.1 Participant information

A total of 23 participants (female = 7) provided both blood samples and fNIRS data in week 6; and 20 of these provided a full data set of three lab visits (week 0, 6, 12) with blood samples and behavioural cognitive testing (female = 6, age median (IQR) = 24 (26) years, age range = 19–58). Of these, nine classified as normal weight (BMI < 25), six as overweight (BMI 25 – 30) and five as obese (BMI > 30). There was no significant change in BMI or pBDNF; sBDNF significantly decreased from week 0 to week 12 (p = 0.005) (Table 1).

There were no significant correlations between pBDNF and sBDNF at any timepoint.

### 3.2 BDNF and cognitive performance

**Fig. 3:**
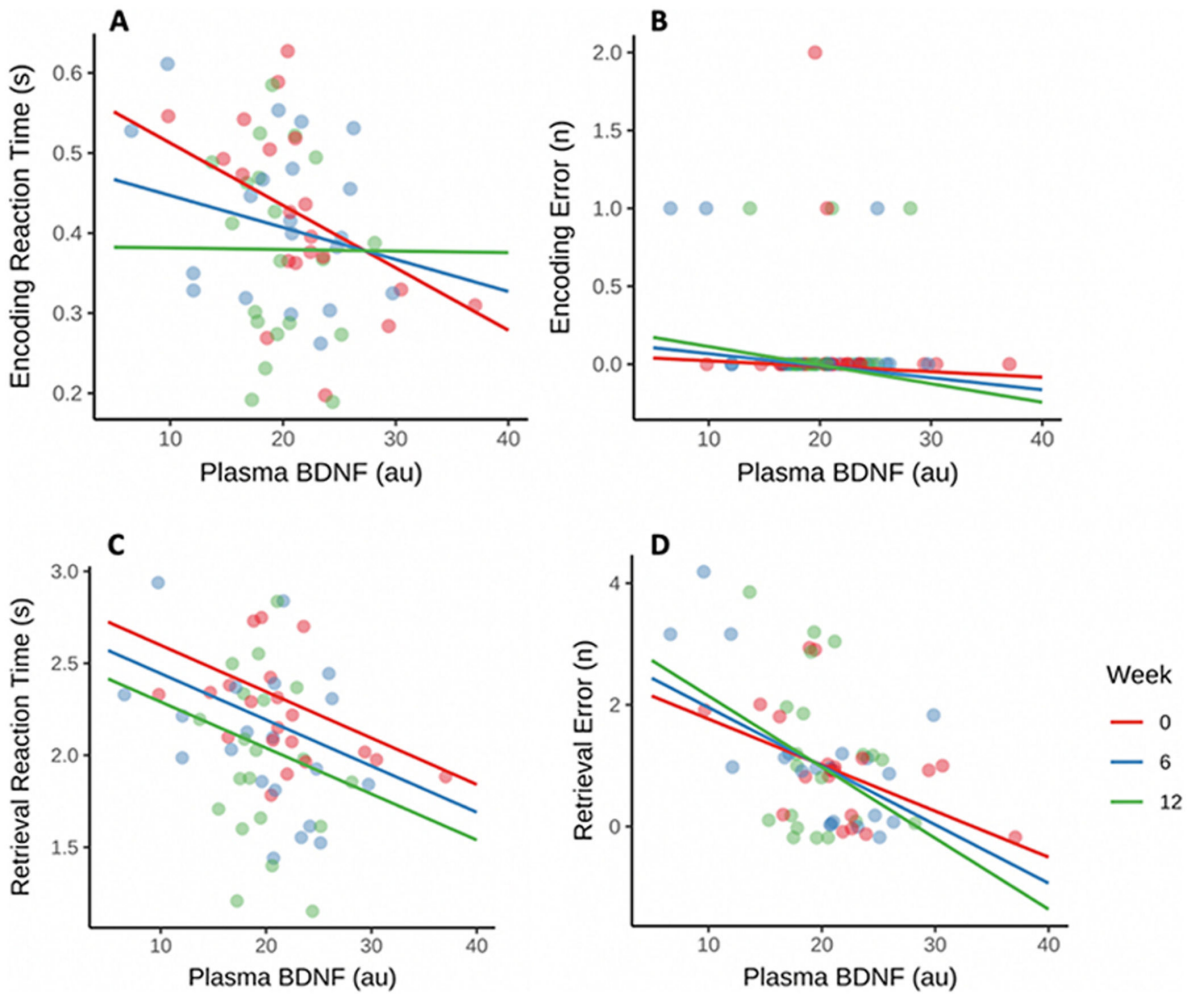
Predictive model outputs for Plasma BDNF (pBDNF) levels on encoding and retrieval task reaction times and errors, for all participants (n = 20). pBDNF values (pg/ml) were Box-Cox transformed, models included random slope and intercept and were age- and sex- adjusted. Lower reaction times and fewer errors both indicate better performance in relation to higher pBDNF for A) encoding reaction times (p = 0.005), D) retrieval reaction times (p = 0.04) and C) retrieval errors (p = 0.05), see Table 2. All models were adjusted for age and sex.

Higher pBDNF, but not sBDNF, was significantly associated with better performance on the memory tasks (p < 0.05) (Fig. 3). Specifically, pBDNF predicted consistently better performance on the retrieval task, with both faster reaction times and fewer errors, with no effect of week (Table 2, Fig. 3C,D). pBDNF also predicted faster reaction times in the encoding task, but no difference in encoding errors (Fig. 3A,B). The full models (adjusted for age and sex) explained 72 % of the variance for encoding reaction times, 49 % and 27 % of the variance in retrieval reaction times and errors respectively (Table 2). There was no association between pBDNF levels and performance on executive function tests (Supplement 3).

There was no association between sBDNF levels and performance on any memory or executive function tests.

### 3.3 BDNF and brain fNIRS

**Fig. 4:**
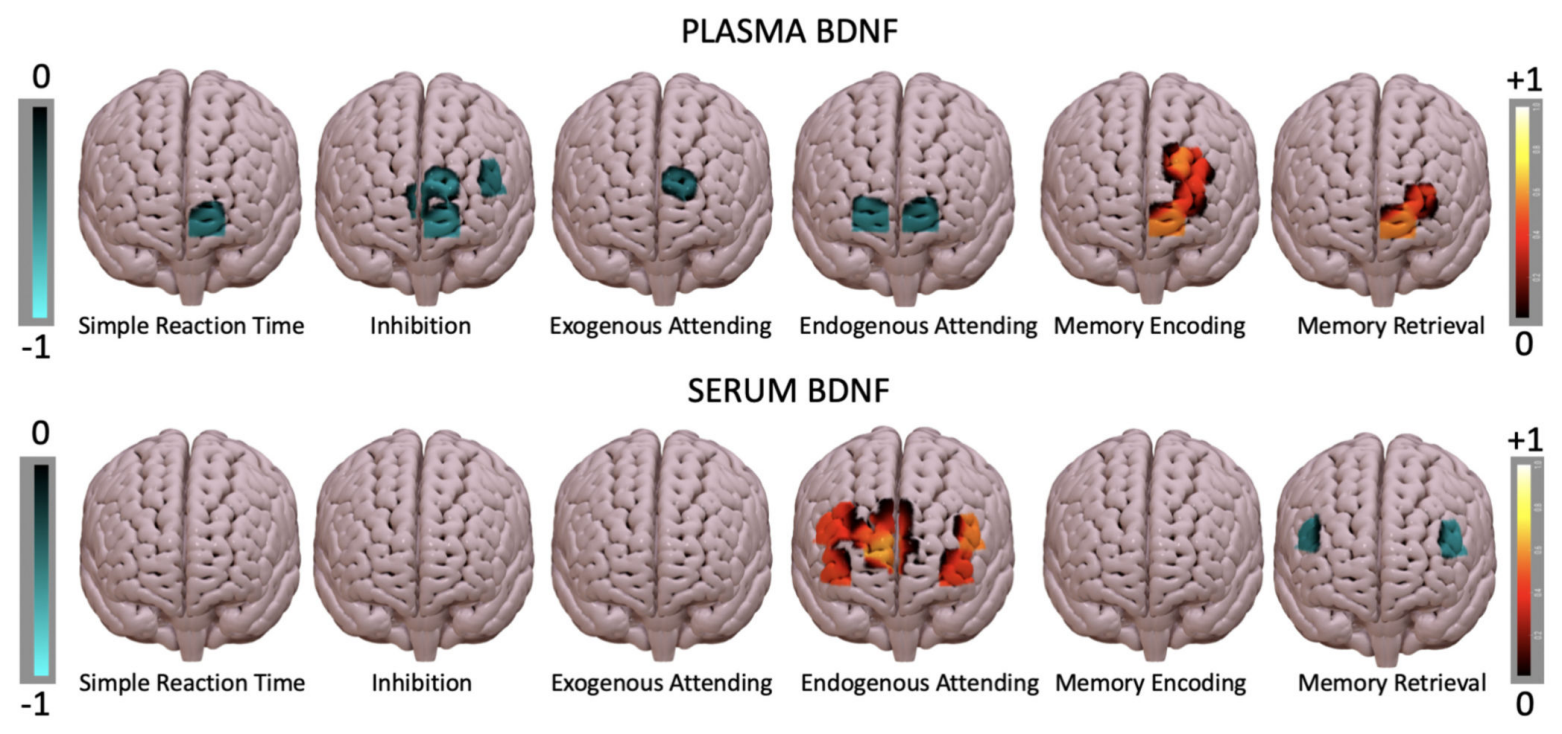
Correlations between pBDNF / sBDNF and CBSI beta values during fNIRS imaging in week 6 (n = 23). Only significant R values (p < 0.05) from the correlations are plotted per channel and per task separately; red = positive R, blue = negative R.

In the fNIRS data, pBDNF correlated significantly (p < 0.05, uncorrected) with CBSI beta values in the left orbitofrontal and frontopolar regions, but the direction of these relationships ran in opposite directions between the attention and inhibition tasks, and the episodic memory tasks (Fig. 4). This pattern was not observed for sBDNF.

**Fig. 5:**
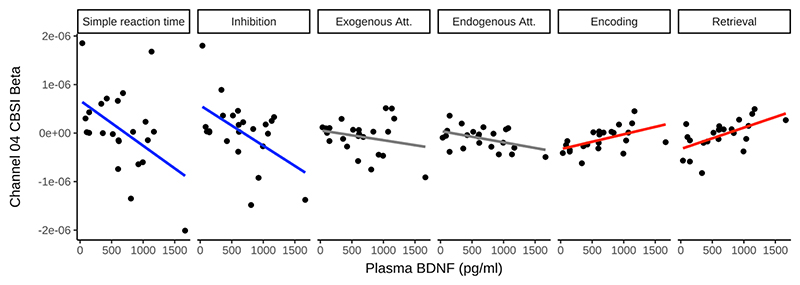
Correlations between pBDNF and CBSI beta values, for Channel 04 only, located across the frontopolar (BA10, 48 % overlap) and orbitofrontal (BA11, 52 % overlap) prefrontal cortex. Simple reaction time (R = −0.46, p = 0.03), Inhibition (R = −0.53., p = 0.009), Exogenous attending (R = −0.22, p = 0.32), Endogenous attending (R = −0.44, p = 0.04), Encoding (R = 0.55, p = 0.007), Retrieval (R = 0.58, p = 0.004). A full correlation table for all channels is available in Supplement 4.

Specifically, during the simple reaction time, inhibition, exogenous and endogenous attending tasks, higher pBDNF correlated with lower CBSI beta values in the medial frontopolar (BA10, R range = -0.55 to −0.41; p range = 0.007 to 0.05) and orbitofrontal regions (BA11, R = −0.53 to −0.44; p = 0.009 to 0.04) (Fig. 5). The inhibition task also exhibited a negative correlation with Broca’s area in the left hemisphere (R = −0.41, p = 0.05). In contrast, during the encoding and retrieval blocks of the memory task, higher pBDNF correlated with higher CBSI beta values in the left lateral frontopolar (BA10, R = 0.45 to 0.41, p = 0.03 to 0.05) and orbitofrontal regions (BA11, R = 0.55/.58, p = 0.007/.004) in both blocks; and in the dorsolateral region during the encoding task only (BA46, R = 0.41 to 0.55; p = 0.006 to 0.049). A full correlation table is included in Supplement 4.

In contrast to pBDNF, sBDNF exhibited positive correlations bilaterally throughout the prefrontal cortex during the endogenous attending task, and negative correlations bilaterally in Broca’s area during the retrieval task (Fig. 4). Specifically, during the endogenous attending task, higher sBDNF related to greater CBSI beta values bilaterally in the medial frontopolar and dorsolateral regions (BA10/BA46, R range = 0.60 to 0.43, p range = 0.002 to 0.04), and in Broca’s area (BA45, left R = 0.53, p = 0.009; right: R = 0.33, p = 0.03); these channels were different to the ones that exhibited a negative relationship with pBDNF. During the retrieval task, sBDNF correlated inversely with CBSI beta values in Broca’s area bilaterally (BA45, left R = −0.51, p = 0.01; right: R = −0.44, p = 0.04).

### 3.4 Task-based fNIRS contrasts

For completeness, task contrasts and correlations with cognitive performance are included in the supplementary material (Supplement 5, 6). In brief, the inhibition task elicited higher CBSI beta values in Broca’s area (left) and bordering dorsolateral prefrontal cortex (BA45/46) (p = 0.04, contrasted with simple reaction time); the exogenous attending task elicited higher CBSI beta values in adjacent channels in the medial frontopolar and bordering dorsolateral area (BA9/10) (p < 0.02, contrasted with endogenous attending); while the retrieval task elicited higher CBSI beta values in multiple adjacent channels in the medial frontopolar area and bilaterally in mirroring channels in the orbitofrontal areas (BA10/11) (p < 0.04, contrasted with encoding). A full table of contrast outputs and correlations can be found in the supplementary material.

## 4 Discussion

The relationships between s/pBDNF and prefrontal cortex function demonstrated task-specific and region-specific effects. Higher pBDNF levels predicted better episodic memory scores alongside greater neural activity in the lateral frontopolar, dorsolateral and orbitofrontal regions. During the attention and inhibition tasks, higher pBDNF related to lower activity in the medial frontopolar and orbitofrontal regions with no effects for cognitive performance. In contrast, sBDNF was not related to any cognitive performance scores, but correlated with greater neural activity across most of the prefrontal cortex during the endogenous attending task, and negative correlations with neural activity in Broca’s area during the memory retrieval task. The effects noted were specific to regions relevant for performance in the various cognitive domains being assessed, highlighting the importance of pBDNF in supporting prefrontal cortex function. Of note, one particular channel which was located between the medial rostral and orbitofrontal cortex (BA10/11) demonstrated significant effects consistently across five of the six blocks implemented, potentially suggesting a key role of pBDNF in the function of this specific region.

### 4.1 Episodic memory and plasma BDNF

The positive association found in this study between pBDNF and episodic memory performance is consistent with literature that reports correlations between pBDNF and memory, and with hippocampal volume, in the elderly (Erickson et al., 2010; Komulainen et al., 2008). Here, the results provide additional insight into BDNF’s role in episodic memory encoding and retrieval by demonstrating a direct link with prefrontal cortex activity in healthy adults during such processes.

The regional specificity of these activations to the lateral frontopolar, orbitofrontal and dorsolateral areas is pertinent to cortical regions that are typically implicated in episodic memory (i.e., the frontopolar and dorsolateral prefrontal cortex, BA10/BA46) (Fletcher et al., 1997). The lateralisation to the left hemisphere observed here is also consistent with research that has identified this pattern, particularly during encoding blocks, and bilaterally during the maintenance of episodic memory retrieval mode (Lepage et al., 2000). In the specific context of the source memory task adopted here, such paradigms typically engage the rostral (BA10), lateral (BA8/9) and dorsolateral prefrontal cortex (BA46), particularly during the retrieval blocks (Crum et al., 2024, Turner et al., 2008).

Therefore, higher pBDNF, but not sBDNF, predicted consistently better source memory performance and greater prefrontal cortex activation in the expected regions during both the encoding and retrieval blocks, highlighting the potential role of bioavailable pBDNF in directly supporting activity of the prefrontal cortex during cognitive processes that underpin episodic memory in healthy individuals.

Fletcher et al. (1997) have recognised that, while BA10 and BA46 are most commonly associated with episodic memory, the less consistent reporting of involvement of other prefrontal cortex regions is likely reflective of differences in materials and stimuli being adopted. In this study, a relationship was observed between sBDNF and activity in Broca’s area during the retrieval block, which is consistent with results from Iidaki et al. (2006), who reported a significant role of this region during an old/new recognition task (a sub-component included in our source memory task). Of note, across the entire battery adopted here, Broca’s area was only associated with sBDNF during the two tasks that required the generation of letters (endogenous attending) or words (source memory retrieval). While sBDNF was not predictive of cognitive performance at any timepoint, this association might potentially be related to language-processing functions in participants with elevated sBDNF (i. e., to respectively visualise letters of the alphabet in their correct order, or to recall the descriptive source of the stimuli). Activity in Broca’s area has been related to character memorisation (Yan et al., 2021) and language generation (Magrassi et al., 2015) in the absence of speech, and similar associations with this same region in the context of BDNF polymorphisms have also been reported by Jasińska et al. (2016). Therefore, these results also point towards a potential role of sBDNF in brain regions involved in language generation, although this is merely speculative, and further research would be needed to explore this result further.

### 4.2 Attention and inhibition

Higher pBDNF levels correlated negatively with activations of the medial frontopolar and orbitofrontal regions during the inhibition and attention tasks, and with Broca’s area in the inhibition task, with no differences in cognitive output (where all tasks required rapid unary or binary responses to externally presented stimuli). The regional specificity of the activations observed here is consistent with previous imaging studies, where inhibition (Go/No-Go) tasks are typically associated with activity in the inferior frontal gyrus (Chikazoe et al., 2009), lateral inferior aspects of the frontopolar prefrontal cortex and sometimes also orbitofrontal regions (Zhang et al., 2017); while endogenous and exogenous attending tasks are typically associated with frontopolar activity (Burgess et al., 2007).

While an effect was observed in prefrontal cortex activity during attention and inhibition, there were no associations between s/pBDNF and cognitive outputs. Theoretically, an association with hemodynamics, in absence of behavioural effects, might explain a difference in bioenergetics in the regions required for these functions, possibly underpinned by BDNF’s role in mitochondrial biogenesis, glucose transport, insulin sensitivity (Di Rosa et al., 2021; Marosi and Mattson, 2014; Takeda et al., 2013), regulation of neurotransmission (Oh et al., 2016; Porcher et al., 2018), and in boosting axonal transport (Tosolini et al., 2022), which could be explored in further mechanistic research specific to the prefrontal cortex.

It is worth noting that the endogenous attending task also exhibited greater activation across most of the prefrontal cortex in relation to higher sBDNF. This task may have required some engagement of language processes (to visualise alphabet letters in their correct sequence, Yan et al., 2021), the allocation of attention to internal thoughts (to attend to each imagined letter) and acting on the conclusion derived (providing a binary response) (Burgess et al., 2007). These three processes have been respectively associated with Broca’s area, the lateral frontopolar prefrontal cortex and the medial frontopolar prefrontal cortex (Yan et al., 2021; Magrassi et al., 2015; Burgess et al., 2007), all of which exhibited greater activity during this task in relation to higher sBDNF. However, these effects were also observed in the absence of any differences in cognitive performance.

So far, a link between circulating BDNF, inhibitory control or attention had not yet been explored. In the broader context of executive function, a few behavioural studies have reported faster response times in cognitive flexibility (Slusher et al., 2018) and faster processing speeds (Moriarty et al., 2019) in relation to higher sBDNF. Together, these findings suggest a potential link between BDNF and executive function, but studies have focused on diverse aspects of the topic (i.e., genetic polymorphisms instead of circulating BDNF, or different subdomains of executive function) and collective findings are inconclusive. Here, we did not find a relationship between executive function performance and circulating s/pBDNF in the behavioural data, but uncovered a relationship with brain hemodynamics in regions that were relevant to the tasks performed, pointing to a potential mechanistic role of pBDNF in cerebral flow regulation which should be explored further. These findings might provide a potential underpinning for future translational research in clinical populations, such as psychiatric disorders and neurodegeneration, where the integrity of the prefrontal cortex is implicated in disease progression.

### 4.3 A possible order effect

Above, we interpret the pBDNF effects in the context of cognitive domain. Given that the cognitive tasks were always presented in the same order, Fig. 5 presents a visible trend that could be related to a time-dependent effect in the contributions of pBDNF to the hemodynamic response throughout the battery. This brings to light a possible alternative explanation where the changing trends in correlation from negative to positive could potentially be reflective of a gradual change in metabolic response and utilisation of pBDNF, starting with a more efficient initial response, and leading towards a utilisation of neural reserve due to fatigue at the end of the battery (Fairclough et al., 2018). The improved performance in the episodic memory task, alongside observed activity in related subregions, would contrast this theory, but it would be difficult to rule out such possibility without counterbalancing the task order. Future research could explore if a time-domain effect of pBDNF exists in supporting neural activity throughout a fatigue-inducing battery.

## 5 Limitations

The sample size in this study was smaller than intended. A sensitivity analysis on our results suggests that the study was powered to detect an effect size of 0.27 in the linear models on behavioural data, which demonstrated higher effects than this threshold for the memory tasks. Similar studies have previously included samples of eight (Moriarty et al., 2019) and thirteen participants (Slusher et al., 2018), with a mean sample of 22 participants across 28 BDNF studies with multiple repeated sampling (Dinoff et al., 2017). Reassuringly, the regional brain activations observed here in a sample of 23 participants are consistent with those observed in our previous studies using the same battery of tests on more than one-hundred participants (Crum et al, 2024).

The exploratory nature of this paper involved the implementation of multiple correlations across all channels in order to assess possible effects across the entire prefrontal cortex. For this reason, corrections for multiple comparisons were not employed, and age was not adjusted for in the channel correlations.

## 6 Conclusion

This study adds novel insight into a potential role of circulating BDNF in supporting prefrontal cortex function. Better cognitive performance and greater activation during episodic memory tasks suggest an association between pBDNF and the prefrontal cortex functions that underpin episodic source memory, adding a new perspective on how pBDNF supports memory formation and retrieval. The differences in activations observed during attention and inhibition tasks in relation to s/pBDNF suggest that BDNF might also relate to hemodynamic differences in frontopolar regions that are required to inhibit behaviour and attend to stimuli. However, in the absence of a correlation with test performance, the significance of this finding requires further investigation. Finally, the differential effects observed between pBDNF and sBDNF emphasise the importance of considering these two BDNF pools separately in future research that investigate the haemodynamic and cognitive functions of the brain. Further studies could consider investigating whether intervening to change BDNF levels modulates prefrontal cortex functions.

## Supplementary Material

Appendix

## Figures and Tables

**Table 1:** Mean ± SD (n = 20) serum and plasma BDNF levels for all participants who attended three weeks of testing. ** Significant change from week 0 (p < 0.01).

	Week 0	Week 6	Week 12
BMI	25.9 ± 5.3	25.8 ± 5.0	25.7 ± 4.9
Plasma BDNF (pg/ml)	798 ± 686	646 ± 422	577 ± 300
Serum BDNF (pg/ml)	25040 ± 5781	23928 ± 5757	21932 ± 4147 **

**Table 2:** Output from the linear mixed models for the two episodic memory blocks showing significant effects of pBDNF on memory performance. Models for all other tasks showed no significant effects of s/pBDNF. All models were adjusted for age and sex; cognitive and pBDNF values were BoxCox transformed where appropriate. Models for all other tasks, which yielded no significance, are included in Supplement 3.

Predictor	Estimate	SE	CI (95 %)	P
	*Encoding reaction time*			
pBDNF	−0.01	0.003	−.01 to −0.003	0.005
Week	−0.02	0.009	−.03 to −0.0006	0.048
pBDNF* Week	0.0006	0.0004	−.0002 to .001	0.13
	*Encoding errors*			
pBDNF	−0.004	0.02	−0.03 to −0.02	0.81
Week	0.01	0.05	−0.08 to −0.12	0.78
pBDNF* Week	−0.0007	0.002	−0.006 to −0.004	0.78
	*Retrieval reaction time*			
pBDNF	−0.03	0.01	−.05 to −0.0008	0.04
Week	−0.03	0.04	−.11 to .06	0.53
pBDNF* Week	−0.0002	0.002	−.004 to .004	0.99
	*Retrieval errors*			
pBDNF	−0.08	0.04	−.15 to .005	**0.05**
Week	0.07	0.13	−.19 to .33	0.61
pBDNF*Week	−0.003	0.006	−.02 to .01	0.58

## Data Availability

Data will be made available on request.
