# Supplementary material for: Plasma and serum BDNF differentially relate to fNIRS prefrontal cortex activity during executive function and memory tasks
Source: Brain Res. Author manuscript; Available in PMC 2026 May 28. (PMC7619107; doi:10.1016/j.brainres.2025.149827)
Supplement: Appendix [file EMS213874-supplement-Appendix.pdf]

Appendix A. Supplementary data

Supplementary data to this article can be found online at https://doi.org/10.1016/j.brainres.2025.149827.

Data availability

Data will be made available on request.

References

Angoa-Perez, M., Anneken, J.H., Kuhn, D.M., 2017. The role of brain-derived neurotrophic factor in the pathophysiology of psychiatric and neurological disorders. J. Psychiatry Psychiatric Dis. 1 (5), 252.

Burgess, P.W., Gilbert, S.J., Dumontheil, I., 2007. Function and localization within rostral prefrontal cortex (area 10). Philos. Trans. R. Soc., B 362 (1481), 887–899.

Burgess, P.W., Wu, H., 2013. Rostral prefrontal cortex (Brodmann area 10). Princ. Frontal Lobe Function 524–544.

Chikazoe, J., Jimura, K., Asari, T., Yamashita, K.I., Morimoto, H., Hirose, S., Konishi, S., 2009. Functional dissociation in right inferior frontal cortex during performance of go/no-go task. Cereb. Cortex 19 (1), 146–152.

Choi, S.H., Bylykbashi, E., Chatila, Z.K., Lee, S.W., Pulli, B., Clemenson, G.D., Tanzi, R.E., 2018. Combined adult neurogenesis and BDNF mimic exercise effects on cognition in an Alzheimer's mouse model. Science 361 (6406), eaan8821.

Yan, C., Christophel, T.B., Allefeld, C., Haynes, J.D., 2021. Decoding verbal working memory representations of chinese characters from Broca's area. Neuroimage 226, 117595.

Crum, J., Ronca, F., Herbert, G., Funk, S., Carmona, E., Hakim, U., Burgess, P.W., 2022. Decreased exercise-induced changes in prefrontal cortex hemodynamics are associated with depressive symptoms. Frontiers in Neuroergonomics 3, 806485.

Crum, J., Ronca, F., Herbert, G., Carmona, E., Jones, I., Hakim, U., Burgess, P.W., 2024. Body fat predictive of acute effects of exercise on prefrontal hemodynamics and speed. Neuropsychologia 196, 108805.

Cui, X., Bray, S., Reiss, A.L., 2010. Functional near infrared spectroscopy (NIRS) signal improvement based on negative correlation between oxygenated and deoxygenated hemoglobin dynamics. Neuroimage 49 (4), 3039–3046.

Di Rosa, M.C., Zimbone, S., Saab, M.W., Tomasello, M.F., 2021. The pleiotropic potential of BDNF beyond neurons: implication for a healthy mind in a healthy body. Life 11 (11), 1256.

Dinoff, A., Herrmann, N., Swardfager, W., Lanctot, K.L., 2017. The effect of acute exercise on blood concentrations of brain-derived neurotrophic factor in healthy adults: a meta-analysis. Eur. J. Neurosci. 46 (1), 1635–1646.

Eigsti, I.M., Zayas, V., Mischel, W., Shoda, Y., Ayduk, O., Dadlani, M.B., Casey, B.J., 2006. Predicting cognitive control from preschool to late adolescence and young adulthood. Psychol. Sci. 17 (6), 478–484.

Erickson, K.I., Prakash, R.S., Voss, M.W., Chaddock, L., Heo, S., McLaren, M., Kramer, A. F., 2010. Brain-derived neurotrophic factor is associated with age-related decline in hippocampal volume. J. Neurosci. 30 (15), 5368–5375.

Fairclough, S.H., Burns, C., Kreplin, U., 2018. FNIRS activity in the prefrontal cortex and motivational intensity: impact of working memory load, financial reward, and correlation-based signal improvement. Neurophotonics. ;5(3):035001. doi: 10.1117/1.NPh.5.3.035001. PMID: 30035151; PMCID: PMC6041856.

Fletcher, P.C., Frith, C.D., Rugg, M.D., 1997. The functional neuroanatomy of episodic memory. Trends Neurosci. 20 (5), 213–218.

Friston, K.J., Holmes, A.P., Worsley, K.J., Poline, J.P., Frith, C.D., Frackowiak, R.S., 1994. Statistical parametric maps in functional imaging: a general linear approach. Hum. Brain Mapp. 2 (4), 189–210.

Gejl, A.K., Enevold, C., Bugge, A., Andersen, M.S., Nielsen, C.H., Andersen, L.B., 2019. Associations between serum and plasma brain-derived neurotrophic factor and influence of storage time and centrifugation strategy. Sci. Rep. 9 (1), 9655.

Gilbert, S.J., Frith, C.D., Burgess, P.W., 2005. Involvement of rostral prefrontal cortex in selection between stimulus-oriented and stimulus-independent thought. Eur. J. Neurosci. 21 (5), 1423–1431.

Jasińska, K.K., Molfese, P.J., Kornilov, S.A., Mencl, W.E., Frost, S.J., Lee, M., Landi, N., 2016. The BDNF Val66Met polymorphism influences reading ability and patterns of neural activation in children. PLoS One 11 (8), e0157449.

Kaur, S., Gonzales, M.M., Tarumi, T., Villalpando, A., Alkatan, M., Pyron, M., Haley, A.P., 2016. Serum brain-derived neurotrophic factor mediates the relationship between abdominal adiposity and executive function in middle age. J. Int. Neuropsychol. Soc. 22 (5), 493–500.

Klein, A.B., Williamson, R., Santini, M.A., Clemmensen, C., Ettrup, A., Rios, M., Aznar, S., 2011. Blood BDNF concentrations reflect brain-tissue BDNF levels across species. Int. J. Neuropsychopharmacol. 14 (3), 347–353.

Komulainen, P., Pedersen, M., Hänninen, T., Bruunsgaard, H., Lakka, T.A., Kivipelto, M., Rauramaa, R., 2008. BDNF is a novel marker of cognitive function in ageing women: the DR's EXTRA Study. Neurobiol. Learn. Mem. 90 (4), 596–603.

Leckie, R.L., Oberlin, L.E., Voss, M.W., Prakash, R.S., Szabo-Reed, A., Chaddock-Heyman, L., Erickson, K.I., 2014. BDNF mediates improvements in executive function following a 1-year exercise intervention. Front. Hum. Neurosci. 8, 985.

Lepage, M., Ghaffar, O., Nyberg, L., Tulving, E., 2000. Prefrontal cortex and episodic memory retrieval mode. Proc. Natl. Acad. Sci. 97 (1), 506–511.

Magrassi, L., Aromataris, G., Cabrini, A., Annovazzi-Lodi, V., Moro, A., 2015. Sound representation in higher language areas during language generation. Proc. Natl. Acad. Sci. 112 (6), 1868–1873.

Marosi, K., Mattson, M.P., 2014. BDNF mediates adaptive brain and body responses to energetic challenges. Trends in Endocrinol. Metab. 25 (2), 89–98.

Miranda, M., Morici, J.F., Zanoni, M.B., Bekinschtein, P., 2019. Brain-derived neurotrophic factor: a key molecule for memory in the healthy and the pathological brain. Front. Cell. Neurosci. 13, 472800.

Moriarty, T., Bourbeau, K., Bellovary, B., Zuhl, M.N., 2019. Exercise intensity influences prefrontal cortex oxygenation during cognitive testing. Behavioral Sciences 9 (8), 83.

Oh, H., Lewis, D.A., Sibille, E., 2016. The role of BDNF in age-dependent changes of excitatory and inhibitory synaptic markers in the human prefrontal cortex. Neuropsychopharmacology 41 (13), 3080–3091.

Pedersen, N.H., Tarp, J., Andersen, L.B., Gejl, A.K., Huang, T., Peijs, L., Bugge, A., 2017. The association between serum brain-derived neurotrophic factor and a cluster of cardiovascular risk factors in adolescents: the CHAMPS-study DK. PLoS One 12 (10), e0186384.

Penny, W. D., Friston, K. J., Ashburner, J. T., Kiebel, S. J., & Nichols, T. E. (Eds.). (2011). Statistical parametric mapping: the analysis of functional brain images. Elsevier.

Pillai, A., Kale, A., Joshi, S., Naphade, N., Raju, M.S.V.K., Nasrallah, H., Mahadik, S.P., 2010. Decreased BDNF levels in CSF of drug-naive first-episode psychotic subjects: correlation with plasma BDNF and psychopathology. Int. J. Neuropsychopharmacol. 13 (4), 535–539.

Pinti, P., Scholkmann, F., Hamilton, A., Burgess, P., Tachtsidis, I., 2019. Current status and issues regarding pre-processing of fNIRS neuroimaging data: an investigation of diverse signal filtering methods within a general linear model framework. Front. Hum. Neurosci. 12, 505.

Porcher, C., Medina, I., Gaiarsa, J.L., 2018. Mechanism of BDNF modulation in GABAergic synaptic transmission in healthy and disease brains. Front. Cell. Neurosci. 12, 273.

R Core Team. (2022). R: A language and environment for statistical computing (Version 4.2.0) [Computer software]. R Foundation for Statistical Computing. https://www.R-project.org/.

Sato, T., Nambu, I., Takeda, K., Aihara, T., Yamashita, O., Isogaya, Y., Osu, R., 2016. Reduction of global interference of scalp-hemodynamics in functional near-infrared spectroscopy using short distance probes. Neuroimage 141, 120–132.

Shimada, H., Makizako, H., Doi, T., Yoshida, D., Tsutsumimoto, K., Anan, Y., Suzuki, T., 2014. A large, cross-sectional observational study of serum BDNF, cognitive function, and mild cognitive impairment in the elderly. Front. Aging Neurosci. 6, 69.

Si, J., Zhang, H., Zhu, L., Chen, A., 2021. The relationship between overweight/obesity and executive control in college students: the mediating effect of BDNF and 5-HT. Life 11 (4), 313.

Slusher, A.L., Patterson, V.T., Schwartz, C.S., Acevedo, E.O., 2018. Impact of high intensity interval exercise on executive function and brain derived neurotrophic factor in healthy college aged males. Physiol. Behav. 191, 116–122.

Tachtsidis, I., Scholkmann, F., 2016. False positives and false negatives in functional near-infrared spectroscopy: issues, challenges, and the way forward. Neurophotonics 3 (3), 031405.

Takeda, K., Kermani, P., Anastasia, A., Obinata, Y., Hempstead, B.L., Kurihara, H., 2013. BDNF protects human vascular endothelial cells from TNFα-induced apoptosis. Biochem. Cell Biol. 91 (5), 341–349.

Turner, M.S., Simons, J.S., Gilbert, S.J., Frith, C.D., Burgess, P.W., 2008. Distinct roles for lateral and medial rostral prefrontal cortex in source monitoring of perceived and imagined events. Neuropsychologia 46 (5), 1442–1453.

Van Praag, H., 2009. Exercise and the brain: something to chew on. Trends Neurosci. 32 (5), 283–290.

Van Praag, H., Kempermann, G., Gage, F.H., 2000. Neural consequences of enviromental enrichment. Nat. Rev. Neurosci. 1 (3), 191–198.

Wagner, S., Kayser, S., Engelmann, J., Schlicht, K.F., Dreimüller, N., Tüscher, O., Lieb, K., 2019. Plasma brain-derived neurotrophic factor (pBDNF) and executive dysfunctions in patients with major depressive disorder. World J. Biol. Psychiatry.

Wyser, D., Mattille, M., Wolf, M., Lambercy, O., Scholkmann, F., Gassert, R., 2020. Short-channel regression in functional near-infrared spectroscopy is more effective when considering heterogeneous scalp hemodynamics. Neurophotonics 7 (3), 035011.

Ye, J.C., Tak, S., Jang, K.E., Jung, J., Jang, J., 2009. NIRS-SPM: statistical parametric mapping for near-infrared spectroscopy. Neuroimage 44 (2), 428–447.

Zhang, R., Geng, X., Lee, T.M.C., 2017. Large-scale functional neural network correlates of response inhibition: an fMRI meta-analysis. Brain Struct. Funct., 222, 3973–3990.
